# Self-Care Monitoring of Heart Failure Symptoms and Lung Impedance at Home Following Hospital Discharge: Longitudinal Study

**DOI:** 10.2196/15445

**Published:** 2020-01-07

**Authors:** Ina Thon Aamodt, Edita Lycholip, Jelena Celutkiene, Thomas von Lueder, Dan Atar, Ragnhild Sørum Falk, Ragnhild Hellesø, Tiny Jaarsma, Anna Strömberg, Irene Lie

**Affiliations:** 1 Centre for Patient-Centered Heart and Lung Research, Department of Cardiothoracic Surgery Oslo University Hospital Ullevål Oslo Norway; 2 Department of Nursing Science, Institute of Health and Society University of Oslo Oslo Norway; 3 Clinic of Cardiac and Vascular Diseases, Institute of Clinical Medicine of the Faculty of Medicine Vilnius University Vilnius Lithuania; 4 Center of Cardiology and Angiology, Vilnius University Hospital Santaros Klinikos Vilnius Lithuania; 5 Department of Cardiology B Oslo University Hospital, Ullevål Oslo Norway; 6 Department of Cardiology Oslo University Hospital Ullevål Oslo Norway; 7 Institute of Clinical Sciences Faculty of Medicine University of Oslo Oslo Norway; 8 Research Support Services, Oslo Centre for Biostatistics and Epidemiology, Oslo University Hospital Oslo Norway; 9 Division of Nursing Department of Social and Welfare Studies Linköping University Norrköping Sweden; 10 Division of Nursing Department of Medical and Health Sciences Linköping University Linköping Sweden; 11 Department of Cardiology Linköping University Linköping Sweden

**Keywords:** heart failure, telemedicine, lung impedance, diary, self-care, prospective study

## Abstract

**Background:**

Self-care is key to the daily management of chronic heart failure (HF). After discharge from hospital, patients may struggle to recognize and respond to worsening HF symptoms. Failure to monitor and respond to HF symptoms may lead to unnecessary hospitalizations.

**Objective:**

This study aimed to (1) determine the feasibility of lung impedance measurements and a symptom diary to monitor HF symptoms daily at home for 30 days following hospital discharge and (2) determine daily changes in HF symptoms of pulmonary edema, lung impedance measurements, and if self-care behavior improves over time when patients use these self-care monitoring tools.

**Methods:**

This study used a prospective longitudinal design including patients from cardiology wards in 2 university hospitals—one in Norway and one in Lithuania. Data on HF symptoms and pulmonary edema were collected from 10 participants (mean age 64.5 years; 90% (9/10) male) with severe HF (New York Heart Association classes III and IV) who were discharged home after being hospitalized for an HF condition. HF symptoms were self-reported using the Memorial Symptom Assessment Scale for Heart Failure. Pulmonary edema was measured by participants using a noninvasive lung impedance monitor, the CardioSet Edema Guard Monitor. Informal caregivers aided the participants with the noninvasive measurements.

**Results:**

The prevalence and burden of shortness of breath varied from participants experiencing them daily to never, whereas lung impedance measurements varied for individual participants and the group participants, as a whole. Self-care behavior score improved significantly (*P*=.007) from a median of 56 (IQR range 22-75) at discharge to a median of 81 (IQR range 72-98) 30 days later.

**Conclusions:**

Noninvasive measurement of lung impedance daily and the use of a symptom diary were feasible at home for 30 days in HF patients. Self-care behavior significantly improved after 30 days of using a symptom diary and measuring lung impedance at home. Further research is needed to determine if daily self-care monitoring of HF signs and symptoms, combined with daily lung impedance measurements, may reduce hospital readmissions.

## Introduction

### Background

Self-care is recognized as an important aspect of daily management of heart failure (HF) and recommended in international guidelines [1-3]. Self-care is defined as a process to maintain health through health-promoting practices and managing illness [4]. Self-care has a positive effect on HF prognosis, HF readmission rates, functional capacity, and well-being [1,4-6]. An important aspect of self-care is self-monitoring, which requires patients to observe and recognize changes in symptoms and signs of HF [4]. Many HF patients struggle with recognizing, interpreting, and taking appropriate action if worsening of symptoms occurs [7,8]. Fluid retention (congestion) often develops gradually in HF, and some HF patients only experience few or atypical symptoms [1]. Improved knowledge among HF patients on possible symptoms of fluid retention and knowing when to consult health care professionals may prevent hospitalizations [9]. Moreover, 1 out of 5 older HF patients are readmitted within 30 days after discharge [10]. In these readmitted patients, HF prognosis remains poor [11,12]. Reducing the readmission rate during the first 30 days after hospital discharge is important to decrease the disease burden experienced by patients and informal caregivers and the economic burden on the health care system [11,13].

The 5 most characteristic HF symptoms of fluid retention are shortness of breath, shortness of breath when supine, shortness of breath that awakens the patient during sleep, feeling tired, and ankle swelling [1]. Moreover, comorbidity is common in patients diagnosed with HF, and some comorbid conditions may have similar symptoms as those of fluid retention, for example, shortness of breath caused by chronic obstructive lung disease [14]. HF patients might therefore benefit from tools that aid them in monitoring and managing their postdischarge symptoms, for example, a diary or a fluid monitoring device. Using a symptom diary, HF patients can successfully detect small changes in symptoms [15,16]. Use of a symptom diary has been associated with improved self-care, survival, and quality of life and fewer HF-related hospitalizations [15-18]. Symptom diaries contain instructions for HF patients and a list of various self-reported HF symptoms in addition to space where patients can provide their weight, blood pressure, heart rate, and comments.

Another promising method for self-monitoring is using noninvasive lung impedance devices to measure pulmonary congestion before HF symptoms are recognized by the patient [19,20]. As pulmonary edema or congestion develops, lung impedance decreases.

### Objectives

Knowledge is lacking for self-care approaches that combine the use of a self-reported symptom diary with noninvasive lung impedance measurements. The aim of this study was, therefore, to assess patients’ HF symptoms, lung impedance, and self-care behavior at home for 30 days after hospital discharge. This study sought answers to the following research questions:

1. How feasible is it for patients with HF and their caregivers to measure symptoms and signs daily at home using a diary and a noninvasive device for measuring lung impedance during a 30-day period after discharge from hospital?

2. How do daily HF symptoms and lung impedance change during the 30-day assessment period?

3. How does self-care behavior change when patients use a symptom diary and a noninvasive device to measure lung impedance during the assessment period?

## Methods

### Study Design and Setting

This longitudinal observational design study was conducted from May 2017 to November 2017 using eligible patients discharged from cardiology wards in 2 university hospitals in Norway and Lithuania. Norway is a high-income country ranked as number 28 by the World Bank, and Lithuania is ranked as number 84 [21]. The health care system in Lithuania is a mixed system funded by the National Health Insurance Fund and the state, whereas funding in Norway is provided by public sources [22,23]. The prevalence of HF was 3.09% and 1.71% in Lithuania [24] and Norway [25], respectively.

### Inclusion Criteria

Patients were eligible if they were hospitalized with a primary diagnosis of HF, aged older than 18 years, fluent in Norwegian or Lithuanian, and possessed sufficient cognitive abilities to understand and complete the study protocol. Cognitive abilities were judged by the nurses or cardiologists at the hospital ward. Only patients with New York Heart Association (NYHA) Functional Classification III and IV were included in the study.

### Exclusion Criteria

Patients with severe HF in need of surgical intervention, advanced chronic kidney disease defined as estimated glomerular filtration rate less than 25 mL per min per 1.73 m^2^ [20], a body weight of greater than 150 kg, documented major depression, or short expected survival time were excluded.

### Data Collection and Instruments

The symptom diary comprised 3 components (Table 1) and was self-administered daily at home for 30 days after hospital discharge. Self-reported questionnaires and a clinical examination were administered both at discharge and at the outpatient clinic 30 days later after completion of the at-home data collection period (see Table 1).

**Table 1:** Data collection instruments administered at discharge, at home or at the outpatient clinic.

Data collection instruments		Discharge		Home for 30 days		Outpatient clinic	
**Symptom diary**							
	Memorial Symptom Assessment Scale-Heart Failure		—^a^		x^b^		—
	Lung impedance		—		x		—
	Medication on demand		—		x		—
**Questionnaire**							
	European Heart Failure Self-care Behavior Scale		x		—		x
Clinical examination		x		—		x	

^a^Not applicable.

^b^The x indicates the time and place of data collection.

### Procedure

Candidate participants were identified by cardiologists at the hospital wards. Before discharge, the included participants were instructed on how to use the printed symptom diary, which consisted of spaces to rate symptoms and to write down lung impedance measurements and on-demand medications. Participants and informal caregivers were trained at their home on how to perform lung impedance measurements and about sending the measurements by SMS text messages to a study mobile phone used by the HF study nurse. The HF study nurse was contacted if the participant had problems with bad electrode connections or other technical problems (for example, difficulty sending SMS data from home). Study participants and informal caregivers were instructed on how to recognize HF symptoms and signs through the use of a paper handout with textual explanations, color-coded information related to HF condition, and prominent contact information.

### Symptom Diary

#### Memorial Symptom Assessment Scale-Heart Failure

Participants rated their symptoms using an adapted version of the Memorial Symptom Assessment Scale-Heart Failure (MSAS-HF) [26], which was modified from the original Memorial Symptom Assessment Scale for cancer patients [27]. The MSAS-HF contains a list of 32 symptoms that HF patients might experience during the most recent 7 days. Participants in our study responded first about the *presence* or absence of each listed symptom and then scored the symptom *frequency* on a Likert scale from 1 to 5 (from rarely to all the time). Symptom *severity* was also scored on a Likert scale from 1 to 5 (from mild to extremely), and symptom *distress* was scored on a Likert scale from 1 to 5 (from a little bit to extremely). The total score is the sum of all the symptoms present, with a minimum score of 0 and a maximum score of 32. Symptom burden scores are determined by calculating the mean frequency, severity, and distress of each symptom, with a possible maximum score of 5 for each experienced symptom. The MSAS-HF was used with permission from the developers [28,29]. To our knowledge, no HF patients have ever used this instrument at home for 30 days after hospital discharge. Translation and cultural adoption of the original MSAS-HF from English to Norwegian and from English to Lithuanian were performed using principles of good practice for the translation and cultural adaption process for patient-reported outcomes [30]. A total of 5 nonparticipating HF patients and their spouses in Norway and Lithuania informally evaluated the translated Norwegian and Lithuanian versions of the MSAS-HF for readability and clarity. Internal consistency of the original MSAS-HF had a Cronbach alpha ranging from .73 to .91 [28,29].

#### Measurement of Lung Impedance

**Figure 1:**
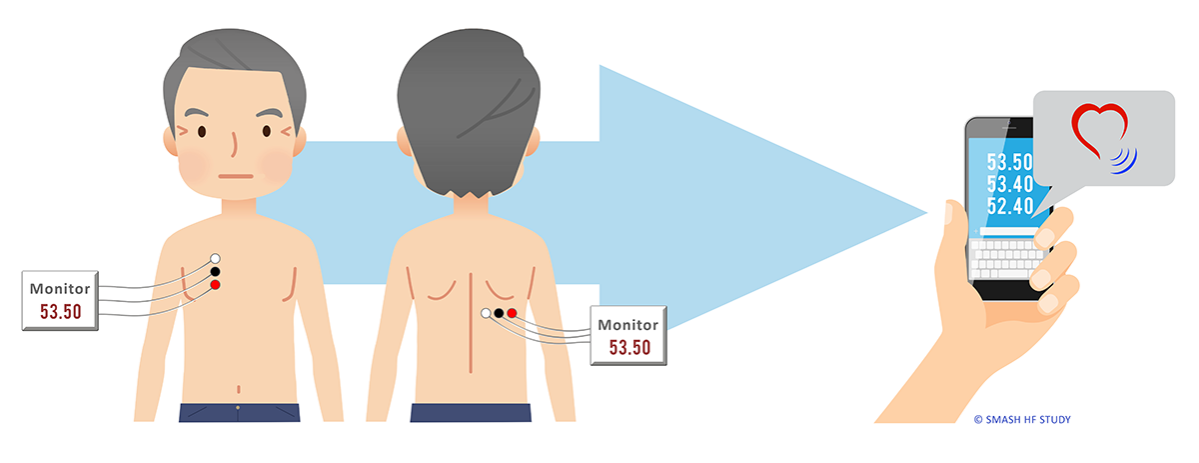
Measurement of noninvasive lung impedance at home and sending impedance data to the study center by SMS. Schematic illustration shows the correct placement of the 6 Edema Guard Monitor electrodes on an HF patient, measurement of impedance, and how daily measurement data are sent by mobile phone (SMS).

The participants and informal caregivers measured lung impedance at home daily using a CardioSet Edema Guard Monitor (Model 0001; CardioSet Medical, Ltd), a noninvasive impedance monitor developed to measure pulmonary congestion or edema in HF patients [20,31]. The patient’s lung fluid status was assessed by the new impedance technique when 100 kHz alternating electrical current was passing through the patient’s chest, and a unique algorithm calculated lung impedance. As HF patients may have pulmonary congestion or edema at discharge, we needed to determine the baseline or *dry* lung impedance of each participant as a reference value. This baseline value was based on each participant’s age, gender, height, weight, NYHA class, and lung impedance measurements [19]. Lung impedance measurement and dry baseline lung impedance values were used to calculate the lung impedance ratio (ΔLIR=[current LI/BLI]−1×100%). Negative ΔLIR values reflect the degree of congestion, with more negative values translating to more congestion. For ΔLIRs between 0% and −18%, change in therapy is not required; however, a larger decrease of −18% to −24% is an indication to optimize treatment. Hospitalization may be necessary if the ΔLIR decreases more than −24% [20]. In this study, if a ΔLIR at discharge was −24% or more and decreased further, the HF study nurse contacted the patients by phone asking about their condition. If necessary, the nurse told the participant to seek help according to the study protocol. Study participants performed the daily measurement in a sitting position each morning with help from their informal caregiver. The caregiver helped place the Edema Guard Monitor electrodes on the same body location every day for the 30 days. A total of 3 lung impedance measurements were taken daily in line with the developer’s recommendations and sent to the HF study nurse by SMS text messages or phone calls. The HF study nurse calculated the mean impedance from the 3 lung impedance values and the ΔLIR each day for every participant. The Edema Guard Monitor consists of 6 electrodes and a device for measurements. The informal caregiver placed the 3 electrodes vertically on the right side of the chest, 4.5 cm from the midline of the sternum, with the upper electrode attached precisely under the clavicle. Next, the informal caregiver placed the 3 remaining electrodes horizontally across the lower edge of the right scapula, with the most leftward electrode located at the crossing point of the horizontal line with the spine [19,20]. Figure 1 shows a schematic illustration of the locations and positions of the electrodes. Here, an HF patient is shown performing the lung impedance measurement and sending the resulting data from home.

#### On-Demand Medication

On-demand medication was prescribed, for example, additional diuretics for some of the included participants to be used at home if necessary. Every day, these HF patients recorded their use of any of these on-demand medications in their diary.

#### Questionnaire

The European Heart Failure Self-care Behavior Scale (EHFScBS) is a self-rating instrument that measures HF-related self-care. It comprises 9 items that are self-scored on a 5-point Likert scale. A standardized score ranges from 0 to 100 results, with higher scores indicating better HF self-care [32-34]. The EHFScBS includes 4 items on consulting behavior and 5 items on other kinds of self-care behavior. The English version of the EHFScBS was translated into Norwegian and Lithuanian [30], as done for the MSAS-HF. Study participants completed the EHFScBS once at discharge and once 30 days later at the outpatient clinic. The EHFScBS-9 item has good internal consistency, having a Cronbach alpha coefficient between .68 and .87 [32]; in this study, the Cronbach alpha coefficient was .87.

#### Clinical Examination, Screening for Depression, and Assessment of Comorbidity

A standard clinical examination was performed at hospital discharge and at the outpatient clinic 30 days later (Table 1) by a cardiologist overseeing the patients care. An HF study nurse noted the patients’ current medication, current NYHA class, and body weight and carefully examined the patients’ jugular vein, ankles, legs, and feet for any signs of HF and fluid retention. Blood was also collected for standard laboratory tests. Participants completed the Hospital Anxiety and Depression Scale questionnaire at discharge to assess the presence of major depression, which would have been exclusionary. The language-appropriate version was used. Major depression was indicated by a score of 11 or greater [35]. Comorbidity at discharge was assessed using the Charlson Comorbidity Index (CCI). The CCI was obtained by patient interview and medical record review [36]. A total of 14 components of comorbidity are presented in the CCI, with severity ranging from value of 1, 2, 3, or 6. HF was not registered as a comorbidity for our participants.

### Data Analysis

EpiData Entry (EpiData Software, 2017) was used for entering and managing data to optimize data accuracy and entry across the 2 countries. This step is in line with recommendation from the data protection managers at both university hospital sites. Raw data were converted from the EpiData Entry format to the SPSS format for analysis. Variables were either categorical or continuous and were presented as counts, percentages, means and SDs, or median and IQRs. Missing data from the symptom diary were not included in the analysis [27] and reported lung impedance data that were collected during an obvious suboptimal electrode connection or lung impedance data for which there was an intraday impedance difference of 3 Ω or more were rejected from analysis [20]. We rejected 2.3% (6/262) of lung impedance measurements for these reasons. Comparison of median self-care behavior at discharge and 30 days later was performed using Related–Samples Wilcoxon signed-rank test. A *P* value <.05 was considered statistically significant [34]. Data were analyzed using SPSS Statistics for Windows, version 25.0 (IBM Corp, released 2017).

### Ethical Considerations

The study was conducted in accordance with the Declaration of Helsinki. Ethical approval was obtained from Regional Committees for Medical and Health Research Ethics in Norway (REC number: 2014/1890) and Vilnius Regional Ethics Committee in Lithuania (approval number: 158200-15-766-280). All participants received verbal and written information about the purpose of the study, signed a written informed consent form before participation, and were free to withdraw from the study at any time. The daily lung impedance measurements were securely sent to the HF study nurse in each country using dedicated study mobile phones. Exchanged study data between the 2 university hospital study sites were deidentified with files encrypted in an email, which required a separate SMS code to be opened. This privacy assurance of research subject data and identity is in accordance with requirements of the data protection officer at the university hospital study sites.

The Edema Guard Monitor lung impedance measuring devices were purchased from the company CardioSet Medical Ltd, Matan, Israel, and researchers and the company signed a written contract with no obligations to the company.

## Results

### Participant Data

A total of 10 participants with HF in NYHA classes III and IV at inclusion (5 from each country) were recruited. The participants’ mean age was 64.5 years, 8 participants had comorbidities, 1 participant was female, and most of the participants lived with their spouse or children (Table 2). None of the participants had self-reported major depression or advanced chronic renal failure. After the 30-day home assessment period, 3 HF patients were classified to be in NYHA class II (at the outpatient clinic). The participants’ demographics and HF characteristics at discharge and after the 30-day period are presented in Table 2.

**Table 2:** Participants’ demographics and clinical characteristics when discharged and 30 days later at the outpatient clinic (N=10).

Characteristics of participants			Discharge		Outpatient clinic
Age (years), mean (range)			64.5 (37-85)		—^a^
**Gender, n**					
	Male	9		—	
**Education,** **n**					
	Less than high school	1		—	
**Informal caregiver at home (help with lung impedance measurement), n**					
	Spouse	7		—	
	Grown children	2		—	
	Nurse	1		—	
**Work status, n**					
	Full time	3		—	
	Retired, disability pension	7		—	
**HF^b^ diagnosis, n**					
	Ischemic HF	7		—	
	Dilated	3		—	
	HF >1 year	7		—	
	HF <1 year	3		—	
**New York Heart Associations classes, n**					
	II	0		3	
	III	8		6	
	IV	2		1	
Implantable cardioverter-defibrillator, n			3		3
Cardiac resynchronization therapy with pacemaker or defibrillation, n			3		3
Jugular venous pressure, n			1		1
Ankle swelling, n			5		2
Systolic BP^c^ (mm Hg), mean (SD)			113.3 (17.5)		119.2 (19.7)
Diastolic BP (mm Hg), mean (SD)			74.7 (13.8)		77.9 (12.4)
Heart rate, mean (SD)			76.5 (13.6)		75.3 (12.0)
**HF medication, n**					
	Angiotensin-converting enzyme inhibitors or angiotensin receptor blockers	9		10	
	Beta blockers	9		9	
	Loop diuretics	10		10	
	Thiazides diuretics	1		1	
	Mineralocorticoid antagonist	7		5	
	Ivabradine	1		2	
	Nitrates	1		1	
**Charlson Comorbidity Index, n**					
	No comorbidity	2		—	
	Low (1-2)	3		—	
	Medium (3-4)	5		—	
**Blood values, mean (SD)**					
	Hemoglobin, g/dL (reference: 13.4-17.0)	14.7 (1.7)		14.6 (1.1)	
	Creatinine, mmol/L (reference: 60-105)	104.3 (23.7)		122.2 (59.4)	
	N-terminal pro b-type natriuretic peptide, pg/mL	2200 (1408)		2910 (2788)	
	Estimate of glomerular filtration rate, mL/min/1.73m^2^	66.3 (19.3)		—	

^a^Number same at discharge and 30 days later at the outpatient clinic.

^b^HF: heart failure.

^c^BP: blood pressure.

### Daily Use of a Symptom Diary and a Lung Impedance Measurement Device at Home

Participants used the symptom diary daily, and with support from their caregivers, they measured lung impedance daily. A total of 7 participants were able to provide data for the full 30-day assessment period. For impedance data, 262 of 300 (87%) recorded measurements were successfully made and sent. For the symptom diary, 1332 of 1500 (89%) entries were successfully made. Missing data were mainly from 2 participants who were classified as NYHA class IV. These participants provided diary and lung impedance data for 19 of 30 days (57%) and 22 of 30 days (63%), respectively. These 2 participants had a few missing lung impedance measurements because of issues with poor signal quality presumably related to suboptimal electrode connections, despite performing more than 3 required measurements and receiving advice from the HF study nurse. A third participant did not have home caregiver support for measuring lung impedance after day 26; thus, participation was terminated on day 26 for that participant. Moreover, 2 participants lived alone, but 1 of them moved in with a family member during the 30-day home assessment period. The other participant received support from an HF study nurse every day to apply the electrodes, although the participant performed the measurement.

### Heart Failure Symptoms During 30-Day Postdischarge Assessment

Prevalence and burden assessment of the 5 symptoms selected from the MSAS-HF varied among the participants (Table 3). Out of the 10 participants, 7 experienced shortness of breath for at least one of the 30 days, and of these participants, 4 experienced it every day of the 30-day period. Out of the 10 participants, 8 reported feeling lack of energy for at least 1 day, and 3 reported lack of energy every day. The least commonly occurring symptom was swelling of hands, legs, or feet. Out of the 10 participants, 3 (30%) reported swelling of the extremities on at least one of the 30 days; no one reported experiencing swelling every day.

The total burden score (mean of the frequency, severity, and distress scores) of *shortness of breath* and *lacking energy* was higher than the total burden score of *difficulty breathing lying flat*; *wake up breathless*; or *swelling of hands, legs, or feet*. Moreover, 1 participant did not experience any of the 5 HF symptoms or signs during the 30-day period. Details of HF symptom prevalence and total burden scores are presented in Table 3.

A total of 9 participants experienced symptoms indicating fluid accumulation for 1 or more days. They reported feeling *shortness of breath* for 5 to 26 days and *lack of energy* for 1 to 28 days.

**Table 3:** The number of participants experiencing (prevalence) and total burden score (mean of the frequency, severity, and distress scores) of the 5 heart failure symptoms and signs during the 30-day postdischarge assessment period (N=10).

Symptom	Prevalence			Total burden score						
	None of the days, n	1 day or more, n	Every day, n	1 day or more			Every day			
				Mean (SD)	Minimum	Maximum		Mean (SD)	Minimum	Maximum
Shortness of breath	3	7	4	2.9 (0.8)	2.3	3.8		2.8 (0.5)	2.3	3.4
Difficulty breathing lying flat	4	6	1	2.4 (1.3)	1.2	4.6		2.8(-^a^)	2.8	2.8
Wake up breathless	5	5	1	1.6 (0.6)	1.0	2.4		2.9 (-)	2.9	2.9
Lack of energy	2	8	3	2.2 (1.3)	1.3	4.4		3.6 (1.1)	2.6	4.8
Swelling of extremities	7	3	0	1.7 (0.6)	1.1	2.3		0 (-)	0	0

^a^Only 1 participant had the symptom every day.

### Noninvasive Lung Impedance Ratio During the 30 Days After Hospitalization

**Figure 2:**
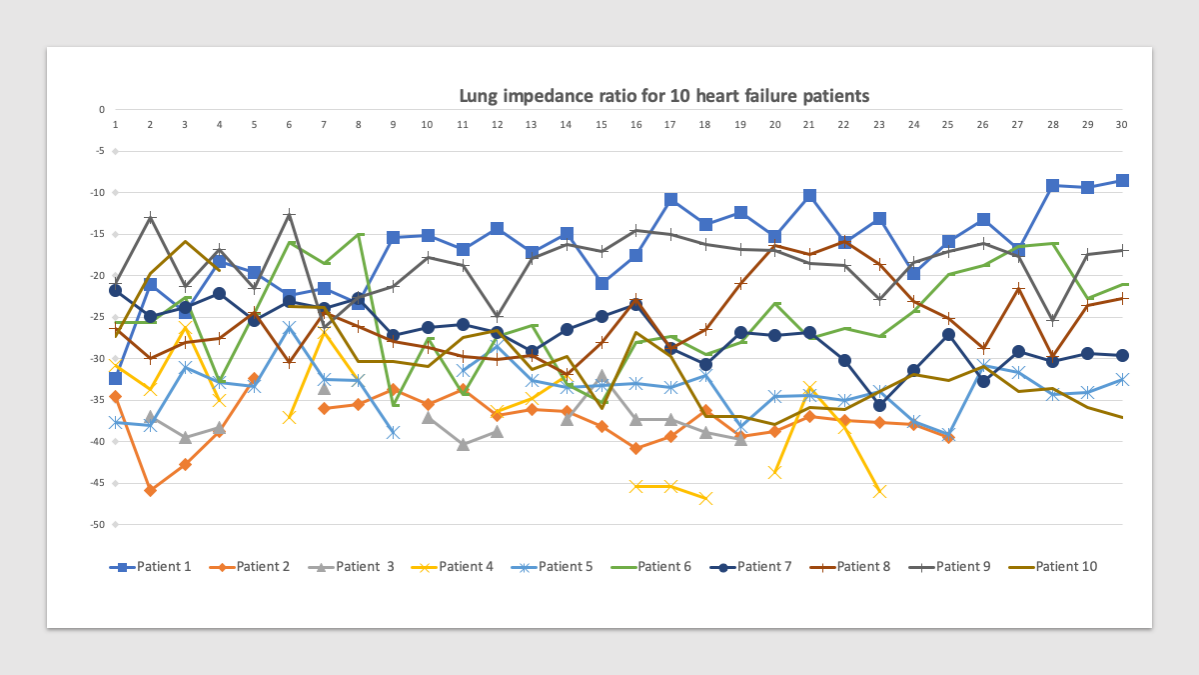
Individual lung impedance ratios (ΔLIRs) during the 30-day postdischarge at-home assessment period. Each color or symbol represents the ΔLIR time series for 1 of the 10 participants. More negative ΔLIRs translate to more pulmonary congestion. Missing data (interrupted time series) resulted from a poor electrode connection, an intervening hospitalization, or a participant temporarily lacking help from their home caregiver to perform measurements. An Edema Guard Monitor (CardioSet Medical) lung impedance device was used to measure lung impedance (see the Methods section for calculation of ΔLIR).

At hospital discharge, individual ΔLIRs ranged from −20.9% to −37.7%. Thus, on day 1 of the study, all participants may have presence of pulmonary edema. The participants’ LIRs over time are presented in Figure 2.

The number of days the patients participated in the self-assessment varied because of technical or health-related reasons. Patient number 2 had 26 days of data, patient number 3 had 19 days of data, and patient number 4 had 23 days of data (Figure 2). Patient number 4 and 10 were temporarily hospitalized during the 30 days because of a worsening HF condition (patient number 4) or acute renal failure (patient number 10). Before readmission, the ΔLIR for patient number 4 was −46.81%, and the ΔLIR for patient number 10 was −19.4%. At the end of the 30-day home assessment period, individual lung impedance values ranged from −8.5% to −39.6%. These values indicate that some participants had minimal pulmonary fluid present (ΔLIRs between 0% and −18%), whereas others had actionable edema requiring adjustment of medication and possible hospitalization. Moreover, 4 patients required medication adjustment during the 30-day study period. Patient number 3 was instructed to self-administer diuretics at home. Patient number 7, 9, and 10 were invited to an outpatient consultation (Lithuanian study site) with a cardiologist for medication adjustment.

### Self-Care Behavior

Self-care behavior improved from the time they were discharged to the end of the 30-day assessment period. The total score on the EHFScBS at the end was significantly better (*P*=.007) than that at hospital discharge. The subscale score on consulting behavior also significantly improved (*P*=.049) after 30 days. At the item level, the most frequently performed self-care behavior (score 5) was *taking medication* *as prescribed*, and the least frequently performed self-care behavior (score 1) was *to exercise regularly*. Details of the self-care behavior results are presented in Table 4.

**Table 4:** Comparison of heart failure participants’ self-care behavior at hospital discharge and 30 days later at the outpatient clinic as assessed with the European Heart Failure Self-care Behavior Scale (European Heart Failure Self-care Behavior Scale-9 comprises 9 items scored on a Likert scale of 1-5, with a higher score indicating better adherence to the given behavior; N=10).

Behavior	Discharge, median (IQR)	30 days, median (IQR)	*P* value
I weigh myself every day	4.5 (1.8-5)	5 (4.8-5)	.04
If my shortness of breath increases, I contact my doctor/nurse	2.5 (1-5)	5 (3-5)	.07
If my feet/legs become swollen, I contact my doctor/nurse	3.5 (1-5)	5 (3.8-5)	.08
If I gain 2 kg in 1 week, I contact my doctor/nurse	2.5 (1- 4.3)	4.5 (2.8-5)	.14
I limit the amount of fluids I drink (<1.5 to 2 L per day)	3.5 (2-5)	4.5 (4-5)	.06
If I experience increased fatigue, I contact my doctor/nurse	2.5 (1.8-3.3)	5 (1.8-5)	.07
I eat a low-salt diet	3.5 (2-5)	5 (4-5)	.03
I take my medication as prescribed	5 (4.8-5)	5 (5-5)	.18
I exercise regularly	2 (1-3)	3.5 (2-5)	.01
European Heart Failure Self-care Behavior Scale, 9 items, total standardized score^a^	56 (22-75)	81 (72-98)	.007
Consulting behavior	55 (38-79)	88 (58-100)	.049

^a^Standardized or summed scores on the European Heart Failure Self-care Behavior Scale, 9 items, ranged from 0 to 100, with higher scores indicating better self-care behavior.

## Discussion

### Principal Findings

Our main finding was that HF patients were able to successfully use a symptom diary and measure aided noninvasive lung impedance at home to self-monitor and report HF symptoms during the 30-day postassessment. This is the first study in which HF patients would use the Edema Guard Monitor to measure their lung impedance on a daily basis for 30 days following hospital discharge to home. Previously, the Edema Guard Monitor had been used by health care professionals once a month at an outpatient clinic with HF patients [20]. Lung impedance measurements were aided by informal caregivers. Study participants significantly improved their self-care behavior during this period. None of the 10 participants or their caregivers had previous experience with the symptom diary or with the noninvasive lung impedance instrument; yet with minimal training, they successfully completed and sent 87% of impedance measurements. Finally, only 2 participants (patient number 4 and 10) had to be readmitted during the 30-day assessment period. Both readmissions were triggered by self-detected worsening conditions (ΔLIR for patient number 4 was −46.81%, and ΔLIR for patient number 10 was −19.4%).

The age range of our participants was 37 to 85 years, and our findings are in line with some reports showing that older patients accept and manage well when using new technology [37]. However, other studies showed that familiarity is key in older patients’ acceptance of new technology at home [38]. All participants in our study had daily contact with the HF study nurse by SMS text messages or phone calls. One reason why our participants managed well may be that they had remote access (phone) to the HF study nurse if they had a poor electrode connection or any other technical problems using the lung impedance device. Another possible explanation is that the participants were actively involved in performing, recording, and sending lung impedance measurements to the HF study nurse. The HF study nurse assessed changes in measurements that participants sent, allowing them to give participants feedback for possible technical adjustments in the device. Participants in other studies likely did not have daily access to professional feedback [39,40]. This kind of access to professional help is not part of routine HF follow-up in Norway and Lithuania. The nurse ratio is different in the 2 countries, with 17.7 per 1000 inhabitants in Norway and 7. 7 per 1000 inhabitants in Lithuania [41].

Typically, after discharge from hospital, HF patients are referred to their general practitioner in the community health care services for follow-up and medication adjustments. In Norway, follow-up at an HF outpatient clinic requires a referral from the hospital ward or general practitioner, currently occurring only 21% of the time [42]. In Lithuania, there is no special follow-up protocol for HF patients. In this study, 3 participants received additional follow-up at the outpatient clinic because they experienced an increase in HF symptoms after medication titration and an improved ΔLIR. Additional follow-up reportedly has a positive impact on the quality of life and self-care of HF patients [43]. Most of our participants also received help from their informal caregivers to measure lung impedance during the 30-day study period. The caregivers expressed positive feelings of being actively involved in the daily measurement of lung impedance and the need for their presence every day [44]. Although some studies report data on caregiver burden from monitoring HF patients [45,46], our study sheds additional light on the caregiver relationship and even reflects a desire to be involved in the care.

Another finding was that participants’ self-reported ratings on the 5 key HF symptoms and on the lung impedance measurements varied greatly. The variability in HF symptoms over the course of the assessment period stresses that constant attention is needed to achieve symptom relief. Some authors also warn that HF patients acclimate to symptoms and adapt their activities and lifestyle to reduce the HF symptoms; thus, they might not recognize these symptoms as warning signs [47-49]. Taken together, these findings indicate that active monitoring is essential for HF patients to build awareness about HF symptoms and signs when confronted with symptoms [49]. Using a symptom diary has the potential to support self-care so that HF patients can play an active role in monitoring their HF symptoms of fluid overload after hospital discharge to home.

HF decompensation is the worsening of HF symptoms and signs and is characterized by a gradual decrease in lung impedance measurements in patients at high risk for volume overload [40]. In this study, participant number 4 exhibited gradual daily changes in lung impedance (Figure 2) and was readmitted to hospital 18 days postdischarge for HF decompensation. Another participant (number 10; Figure 2) similarly experienced a gradual change in lung impedance but was readmitted shortly after discharge for acute renal failure. In our study, gradual changes in HF symptoms appeared relatively quickly following discharge compared with those reported in other studies, which took several weeks to manifest [40]. This may be because, in our study, these patients received adjustments in their medications or because data were missing. Another reason for discrepancy of results across the HF literature relates to use of inconsistent terminology to describe pulmonary edema symptoms and to differing methods used to measure edema (eg, thoracic, intrathoracic, or bioimpedance measurements and noninvasive methods) [39,50-52].

We observed that self-care behavior of the study participants significantly improved after using the daily symptom diary and taking daily lung impedance measurements. At discharge, our participants had inadequate self-care. According to the EHFScBS, a score of 70 or higher reflects adequate self-care behavior [53], which is defined as actions to maintain life, healthy functioning, and well-being [32]. In patients with HF, inadequate self-care is an independent risk factor for adverse clinical outcomes [54]. After the 30-day home assessment period, our participants’ self-care behavior significantly improved, as their EHFScBS scores reached or exceeded the 70-point threshold, suggesting adequate self-care. The professional activities of the HF study nurse could be one reason for this improvement in self-care among our participants. The nurse was responsible for following up each participant daily. This kind of follow-up is not part of usual follow-up for HF patients in either country. Other studies have reported that self-care is troublesome for HF patients after hospital discharge to home (eg, coordinating own care and medications and requiring information and training) [55]. Moreover, HF patients who use invasive lung impedance measuring devices and who report better self-care also have fewer episodes of decrease in lung impedance [56]. Another potential reason for the improved self-care behavior we observed is that our study enabled our participants to establish a new daily routine of filling in a symptom diary and measuring lung impedance. Routines and habits are factors known to positively influence self-care [6]. In addition, the education and the handout summarizing HF warning signs and the emergency contact that the participants received could have contributed to participants’ improvement in consulting behavior. Using tools to support HF patients can, over time, change their self-care behavior for the better. Indeed, in HF patients, regular assessments are encouraged [57]. This study has demonstrated that using new self-care tools, an HF symptom diary, and a noninvasive impedance device, during the first 30 days after hospital discharge can contribute positively to better self-care.

### Strengths and Limitations

One limitation is that only 10 patients participated in this study. Nonetheless, we were able to obtain a substantial amount of data from these participants, and these data provided us with an in-depth understanding of the trajectory of HF symptoms during the first month at home after hospitalization. The variability of the data for HF symptoms and LIR, in addition to our small sample, made it not advisable to perform an analysis for possible associations among these variables. Another limitation is the use of an unvalidated version of the MSAS-HF for the purpose of daily self-monitoring for 30 days at home. However, the unvalidated version did include 5 HF symptoms of fluid overload that are also recommended in guidelines [1]. Moreover, before our study, the MSAS-HF had never been used every day for 30 days by HF patients after hospital discharge. Thus, these data represent pilot normative data for the daily use of MSAS-HF.

### Conclusions

This is the first study showing that discharged HF patients can successfully use a symptom diary combined with measurement of their noninvasive lung impedance at home to self-monitor on a daily basis, with support from informal caregivers. We found a significant improvement in the participants’ self-care behavior in the period between hospital discharge and end of the 30-day home assessment. These self-care tools can be used by nurses in clinical practice to educate HF patients and their informal caregivers before discharge to home, which could also reduce readmissions. For future research, a larger study population is needed to determine whether self-monitoring of particular HF symptoms at home and LIR are predictive of HF deterioration. Early detection of worsening HF at home will aid health care professionals in providing better HF care.

## Abbreviations

CCI: Charlson Comorbidity Index
EHFScBS: European Heart Failure Self-care Behavior Scale
HF: heart failure
LIR: lung impedance ratio
MSAS-HF: Memorial Symptom Assessment Scale-Heart Failure
NYHA: New York Heart Association
